# Electrospun Fibre Webs Templated Synthesis of Mineral Scaffolds Based on Calcium Phosphates and Barium Titanate

**DOI:** 10.3390/nano10040772

**Published:** 2020-04-16

**Authors:** Cristina Busuioc, Elena Olaret, Izabela-Cristina Stancu, Adrian-Ionut Nicoara, Sorin-Ion Jinga

**Affiliations:** 1 Science and Engineering of Oxide Materials and Nanomaterials Department, University Politehnica of Bucharest, 1-7 Polizu Street, District 1, RO-011061 Bucharest, Romania; cristina.busuioc@upb.ro (C.B.); adi.nicoara18@gmail.com (A.-I.N.); 2 Advanced Polymer Materials Group, Faculty of Applied Chemistry and Materials Science, University Politehnica of Bucharest, 1-7 Polizu Street, District 1, RO-011061 Bucharest, Romania; olaretelena@gmail.com (E.O.); izabela.cristina.stancu@gmail.com (I.-C.S.)

**Keywords:** fibre webs, electrospinning, calcium phosphates, barium titanate, mineral scaffolds, bone engineering

## Abstract

The current work focuses on the development of mineral scaffolds with complex composition and controlled morphology by using a polymeric template in the form of nonwoven fibre webs fabricated through electrospinning. By a cross-linking process, gelatine fibres stable in aqueous solutions were achieved, these being further subjected to a loading step with two types of mineral phases: calcium phosphates deposited by chemical reaction and barium titanate nanoparticles as decoration on the previously achieved structures. Thus, hybrid materials were obtained and subsequently processed in terms of freeze-drying and heat treating with the purpose of burning the template and consolidating the mineral part as potential bone implants with improved biological response by external stimulation. The results confirmed the tunable morphology, as well as the considerable applicability of both as-prepared and final samples for the development of medical devices, which encourages the continuation of research in the direction of assessing the synergistic contribution of barium titanate domains polarisation/magnetisation by external applied fields.

## 1. Introduction

The field of tissue engineering has grown considerably in recent years, one of the driving forces for such an evolution being the exploration and exploitation of composite biomaterials, which ensure superior performances compared to the unitary counterparts [1,2]. In this way, the inherent disadvantages of some materials with demonstrated potential in the medical devices industry were suppressed by combining them with other phases, which made possible the revealing of derivatives with improved properties [3,4] or multifunctional systems [5,6].

Thus, ceramic and polymeric representatives constitute a real resource pool for designing and developing implants dedicated to hard tissue replacement or reconstruction [7]. Starting from the theoretical premises of composition and microstructure related to the natural bone, namely calcium and phosphorus containing mineral phases and specific sponginess that ensure both light weight and adequate mechanical strength [8], a wide range of associations between different types of calcium phosphates and polymers have been proposed [9,10,11]. Usually, the spongy appearance is artificially generated through the use of porogenous agents [12,13], dedicated techniques [14,15], or sacrificial templates [16,17,18]; further, the size, shape, distribution, and interconnection of the voids must be engineered so as to favour the natural processes from the implant–living tissue interface [19]. Another territory with huge potential is represented by the development of smart biomaterials, which allow an external stimulation of the physiological microenvironment, offering beneficial conditions for the cellular metabolism. In this respect, different materials with electrical or magnetic properties have been integrated into older efficient systems or processed as novel devices for the field of hard tissue engineering [20,21,22].

Among the broad variety of techniques dedicated to the production of porous three-dimensional structures, electrospinning is the most approached due to its simplicity, but also because it generates materials with a large surface-to-volume ratio and suitable microstructure for bone implants [23,24]. Even though most of the products are of organic nature [25,26], there are also examples of electrospun ceramic webs [27]. Moreover, biopolymers have become extremely attractive lately due to their biocompatibility, biodegradability, and low cost, which are features that led to various medical applications, such as encapsulation and the release of bioactive molecules, reinforcing agents for different matrices, or diagnostic and therapeutic imaging [28,29]. In this context, gelatine is a protein of natural origin that is obtained by collagen hydrolysis; briefly, it is a biopolymer that meets all the requirements imposed in the field of tissue engineering [30,31,32]. On another hand, calcium phosphates are of great interest due to their biocompatibility and bioactivity [33,34,35], the most known representatives being hydroxyapatite (Ca_10_(PO_4_)_6_(OH)_2_), dicalcium phosphate dihydrate (CaHPO_4_·2H_2_O), octacalcium phosphate (Ca_8_H_2_(PO_4_)_6_·5H_2_O), dicalcium phosphate (CaHPO_4_), tricalcium phosphate (Ca_3_(PO_4_)_2_), tetracalcium phosphate (Ca_4_O(PO_4_)_2_), and calcium pyrophosphate (Ca_2_P_2_O_7_) [36]. Thus, the fabrication of composite fibres seems to be a solution for many ongoing medical issues [37]. K. Chan et al. [38] studied the combination of polylactic acid with different oxide ceramics, since such electrospun composite scaffolds can provide osteogenic properties, while D. Santos et al. [39] demonstrated that an electrospun membrane of polylactic acid with glass-reinforced hydroxyapatite microparticles represents an environment that enhances cell proliferation. M.K. Ahmed et al. [40] reported the fabrication through the electrospinning of composite scaffolds based on polycaprolactone microfibers with alumina and selenite-doped carbonated hydroxyapatite content, showing that a variable ratio between the two ceramic additives allows properties’ tuning. In order to surpass the infections complication in orthopaedic surgery, Liu et al. [41] proposed a complex system based on polylactic acid fibres, hydroxyapatite nanowires, polydopamine adhesive, silver nanoparticles, and a polypyrrole mediator. Other approaches consist of coating the electrospun polymeric fibre webs with metallic [42] or ceramic [43] layers to increase the hydrophilicity, biocompatibility, or bioactivity, as well as employing the electrospun membrane as a template [44] to retain the primary microstructure.

Moving to the possibility of local stimulation at the affected area, this can be achieved by using piezoelectric, ferroelectric, or ferromagnetic materials that are distributed in a controlled manner and activated by the application of external electric or magnetic fields, subsequently improving the speed and quality of tissue regeneration by modifying the biological routes travelled by the cells [45]. One such example is barium titanate (BaTiO_3_), which is a well-known ceramic with unique properties [16,46,47,48,49]. It was demonstrated that the integration of hydroxyapatite and barium titanate powders in a collagen matrix can improve the osseointegration process of the resulting hybrid scaffolds [47], the influence of barium titanate being afterwards evaluated through numerical simulation [46]. Composite scaffolds based on calcium phosphates and barium titanate were also manufactured by bacterial cellulose templated synthesis, being well accepted by the mesenchymal stem cell cultures [16].

In conclusion, the microstructure of a bone substitute must meet the requirements related to the macropores’ proportion and their mode of assembly so as to allow a facile penetration of cells and ensure the local integrity when subjected to medium and heavy loads. As a result, the present work proposes a new route for obtaining porous three-dimensional structures based on calcium phosphates and barium titanate, this being the first attempt of this kind in the field. The current technological flow is based on the use of an electrospun fibre web as a template and porogenous agent simultaneously, which is loaded/decorated with mineral phases by chemical synthesis in solution or physical attachment from suspension and subsequently removed from the system by heat treatment.

## 2. Materials and Methods

### 2.1. Fibrous Template Production

Firstly, a fibrous fish gelatine (*Gel*) web was fabricated through the electrospinning technique (Electrospinning Apparatus EC-CLI, IME Technologies). Basically, a 70% *w*/*v* fish *Gel* precursor solution was prepared by dissolving the corresponding amount of protein in double-distilled water under continuous stirring in a water bath at 40 °C for at least 4 h. The obtained solution was loaded in a 5-mL syringe after reaching room temperature (25 °C) and subjected to the electrospinning process, according to the following parameters: 7 μL/min feeding rate, 21 kV applied voltage, 5 mm/s needle moving speed, and a 70 rpm collector (2 cm diameter) rotating speed, respectively. The equipment is provided with an environmental control cabinet, which kept the temperature (25 °C) and relative humidity (40%) constant during the entire fabrication process. Secondly, the detached *Gel* fibre web was stabilised against aqueous media by a cross-linking process. In this respect, the fibrous substrate was incubated in a 0.5% glutaraldehyde ethanolic solution for 4 days at 25 °C and washed for additional 5 days, as follows: 3 days rinsing with ethanol and 2 days rinsing with double-distilled water. The obtained fibre template was kept at 4 °C before further use.

Such fibrous substrates can be integrated in a wide range of medical applications, as previously reported: selective separation, the immobilisation of active agents, wound healing, artificial skin, drug delivery, scaffold for tissue engineering, nervous system, and bone tissue engineering [50].

### 2.2. Hybrid Composites Synthesis

In the second step, the template with a fibrous three-dimensional structure was loaded with calcium phosphates (CPs), resulting in a hybrid composite consisting of a network of randomly distributed polymeric fibres covered with a layer of CPs having different compositions, which are most likely a combination between brushite (CaHPO_4_·2H_2_O) as a major phase and hydroxyapatite (Ca_10_(PO_4_)_6_(OH)_2_) as a minor phase [51]. A deposition cycle involved the mineral phases loading onto the template, which was cut into pieces of 1.5 × 1.5 cm^2^ by a chemical reaction in liquid medium. First, the electrospun fibre webs were immersed in 100 mL 0.5 M calcium nitrate tetrahydrate (Ca(NO_3_)_2_·4H_2_O, Merck) aqueous solution, with pH adjusted to 10–11 with an ammonium hydroxide (NH_4_OH) aqueous solution and maintained under ultrasonication for 15 min. Then, they were removed and immersed in 100 mL 0.5 M ammonium phosphate dibasic ((NH_4_)_2_HPO_4_, Merck) aqueous solution for another 15 min under ultrasonication. This two-step synthesis represents a deposition cycle; for several cycles, the immersion procedures in the solutions containing Ca^2+^ and PO_4_^3−^ were repeated in the same order. The samples were coded *Gel*-CPs-1c in the case of applying one deposition cycle and *Gel*-CPs-3c for three such cycles.

The third step involved the decoration of the hybrid composite obtained in the previous stage with barium titanate (BT) nanoparticles synthesised by combining the sol–gel method with a hydrothermal treatment [48]. Thus, the dried gel, formed following the hydrolysis and polycondensation processes, was autoclaved at 120 °C for 24 h, filtered, and dried, resulting in a white powder. This was added to (NH_4_)_2_HPO_4_ solution in the amount of 0.5 g, and the same protocol was followed with the aim of ensuring both loading with CPs and decoration with BT nanoparticles. The attained sample is further referred as *Gel*-CPs-BT-3c, taking into account the fact that BT was associated only with the three cycles derived composites, since usually a larger amount of material is needed to ensure the formation of a self-sustained structure.

The as-prepared samples were freeze-dried (freeze-drying process under standard conditions) to preserve the porous three-dimensional structure of the template and obtain spongy composite materials. The advantages of the present route consist of the high purity and homogeneity of CPs deposition due to the ionic contact between reactants in liquid medium; the tetragonal crystalline symmetry of BT nanoparticles due to the applied hydrothermal treatment, which could provide a measurable response to external stimuli, so that the physiological microenvironment could become more attractive to the cells; and nanoscale mixing of the constituent phases due to the application of ultrasounds to facilitate the penetration of the precursor solutions/suspension in the volume of the electrospun fibre web.

### 2.3. Mineral Scaffolds Fabrication

**Figure 1:**
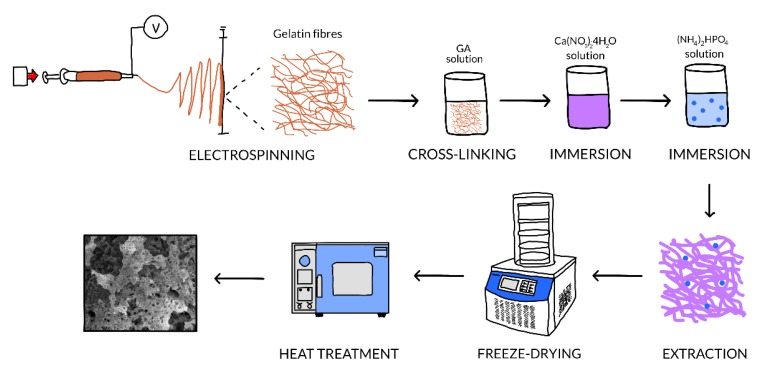
Schematic representation of the technological flow.

In the last step, the composites described above (*Gel* fibre webs-CPs layer-BT nanoparticles) were heat treated in air, under different experimental conditions, in order to remove the polymeric template by combustion, complete the crystallisation of CPs, and generate mineral scaffolds with various morphologies. Simultaneously, the achievement of a mechanical resistance suitable for bone substitute applications was targeted. More exactly, the selected values of the heat treatment parameters for obtaining porous three-dimensional structures were a heating rate of 60 °C/h up to 300 °C, 120 °C/h up to 600 °C, and 600 °C/h up to the maximum temperature, 800 °C, 1000 °C, or 1200 °C maximum temperature, and a 2 h period at maximum temperature (Figure 1). A reference is made to the obtained samples by mentioning the temperature at which they were processed (800 °C, 1000 °C, and 1200 °C).

The current approach is characterised by its use of cheap and widely available materials, as well as simple and accessible techniques, while the structural, morphological, and biological properties of the final materials can be adapted to the clinical particularities of each patient, with a significant reduction of the healing period and improvement of the life quality during recovery.

### 2.4. Materials Characterisation

The samples were characterised from the material point of view: thermal, compositional, structural, and morphological. The thermal analysis was conducted from room temperature to 1000 °C, 5 °C/min temperature rate, in air, on a Shimadzu DTG-60 equipment (Shimadzu Corporation, Kyoto, Japan). The mineralogical composition and crystalline structure were evaluated by X-ray diffraction (XRD), with a Shimadzu XRD 6000 diffractometer (Shimadzu Corporation, Kyoto, Japan) with Ni-filtered Cu *K*α radiation (*λ* = 1.54 Å), 2*θ* ranging between 10 and 80°, a 0.02° step size, a 2°/min scan speed, and 0.6 s preset time. The vibrational features were analysed by Fourier transform infrared (FTIR) spectroscopy, with a Thermo Scientific Nicolet iS50 spectrophotometer (Thermo Fisher Scientific, Waltham, MA, USA), using the attenuated total reflection (ATR) module, the wavenumber ranging between 400 and 4000 cm^−1^, 32 scans per sample, and 4 cm^−1^ resolution, as well as by Raman spectroscopy, with a Horiba Confocal LabRAM HR Evolution spectrophotometer (Horiba, Kyoto, Japan), using a 633 nm laser, 100% ND filter, 50 × objective, and 600 gr/mm grating, the wavenumber ranging between 200 and 1000 cm^−1^, 10 scans per sample, and 10 s per scan. The morphology was revealed by scanning electron microscopy (SEM), with a Thermo Scientific Verios G4 (Thermo Fisher Scientific, Waltham, MA, USA) (for barium titanate powder; 2 kV accelerating voltage, 2 mm working distance and no metallic coating) or Quanta Inspect F microscope (FEI Company, Hillsboro, OR, USA) equipped with an energy-dispersive X-ray spectroscopy (EDX) probe (for all the other samples; 30 kV accelerating voltage, 10 mm working distance, and gold coating by DC magnetron sputtering for 60 s).

## 3. Results and Discussion

**Figure 2:**
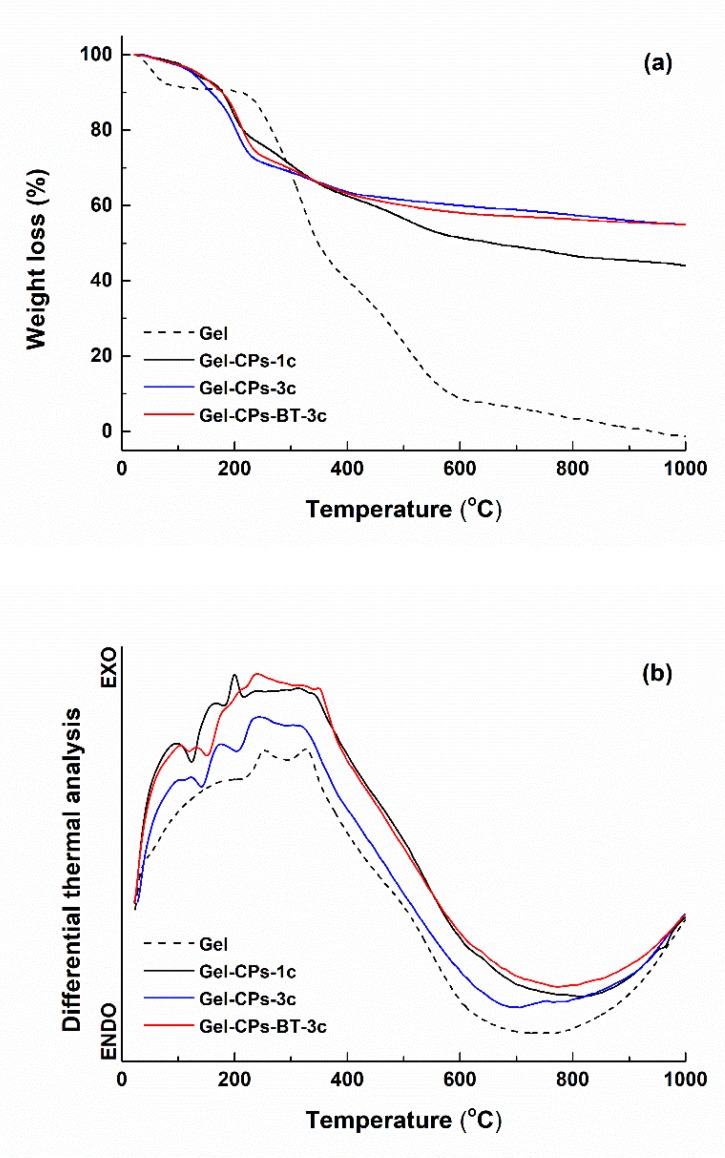
Complex thermal analyses of the template and as-prepared composites: (**a**) weight loss and (**b**) differential thermal analysis.

The bear and loaded fibre webs were characterised regarding their thermal behaviour when heated from room temperature up to 1000 °C (Figure 2). As it was expected, the *Gel* sample is completely burnt at the final temperature, with 100% weight loss, this being divided in several steps, with the major one occurring at approximately 320 °C (protein thermal degradation or decomposition); this value is a little bigger than those previously reported (250–300 °C) [52], which is an aspect that can be correlated with the cross-linking procedure and higher degree of crystallinity that the electrospun fibre web displays (Figure 4a). The shape of the weight loss curves (Figure 2a) changes when it comes to the composite samples, with a significant difference in the final weight loss between the one and three cycles of derived materials: 56% for *Gel*-CPs-1c and 45% for *Gel*-CPs-3c and *Gel*-CPs-BT-3. These values indicate at least two findings: the application of multiple deposition cycles is not a linear process of increasing the loading quantity and the developed protocol generates reproducible samples, since the value is identical for the two specimens obtained after applying three deposition cycles (the amount of BT decoration is reduced and does not modify the overall trend). Moreover, in these three last cases, the major weight loss shifts to 210 °C, which is related to the loss of adsorbed, absorbed, and bounded water, while the core fibres seem to be somehow protected by the mineral shell, pushing the degradation processes to higher temperatures. Thus, depending on the number of applied deposition cycles, the degree of loading with mineral phases can be controlled so that the fibre coating layer reaches the desired thickness (Figure 5b,c). The proposed phenomena are also sustained by the thermal effects shown in Figure 2b: a succession of endothermic processes at small temperatures, followed by several exothermic processes above 220 °C.

**Figure 3:**
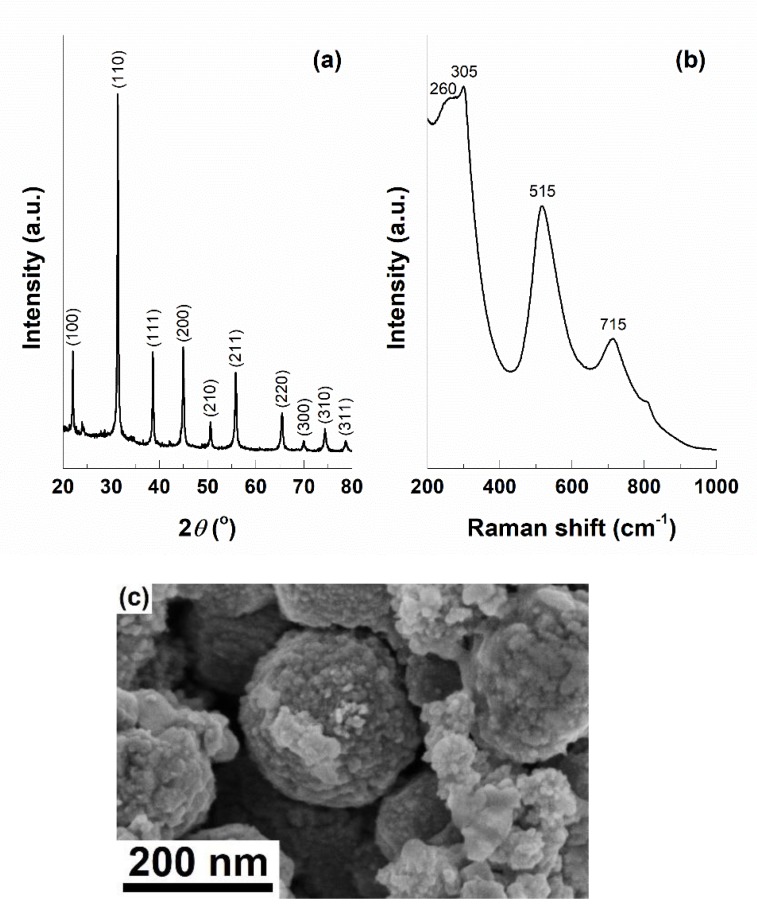
(**a**) XRD pattern, (**b**) Raman spectrum and (**c**) SEM image of barium titanate (BT*)* powder.

Before integrating the BT phase within the composite system, it was investigated separately in order to reveal its crystallinity and morphology. Figure 3a presents the corresponding XRD pattern, which indicates the obtaining of the perovskite structure with cubic symmetry (ICDD 04-007-6869). However, the powder was also studied by Raman spectroscopy in order to identify possible differences in symmetry compared to the former diffractogram, because the dried gel was autoclaved and, in this way, it was subjected to high pressure. The resulting Raman spectrum (Figure 3b) contains four vibrational bands, from which the one at 305 cm^−1^ is the most important, since it is correlated with the existence of a tetragonal symmetry [47,53,54]. This apparent contradiction can be explained based on the distortion of the cubic structure into a pseudo-cubic or slightly tetragonal one. Such an assertion, together with the emergence of oxygen vacancies at the particles surface, trigger peculiar magnetic properties, namely an unexpected ferromagnetism at room temperature; thus, Ti^3+^ or Ti^2+^ ions appear, offering a non-zero magnetisation below a critical nanoparticle size, as well as a multiferroic behaviour [48,55,56]. This phenomenon can be subsequently exploited in the direction of local stimulation of the cell physiological microenvironment. Figure 3c exhibits an SEM image of the same powder, emphasising individual spherical particles with dimensions below 10 nm but gathered as rounded aggregates of few hundreds of nm.

**Figure 4:**
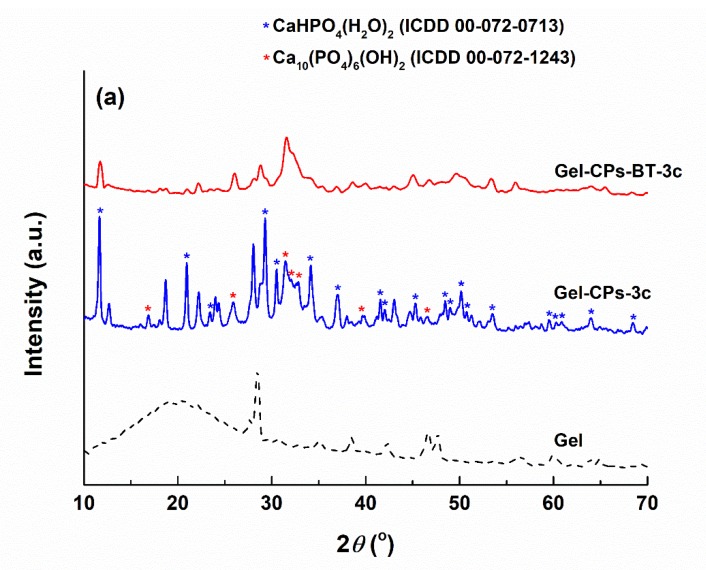

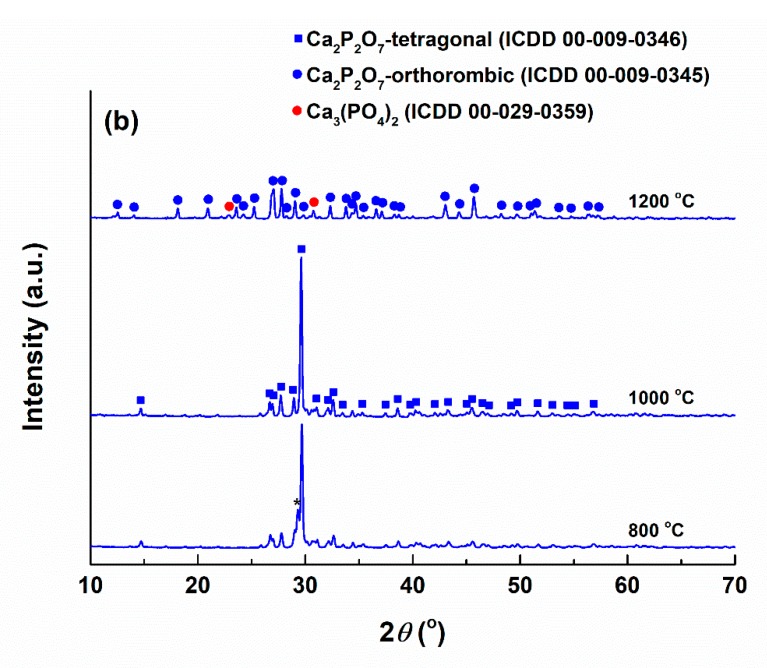
XRD patterns of the obtained materials: (**a**) before the thermal treatments and (**b**) after the thermal treatments of the *Gel*-CPs-3c composite.

The XRD patterns of the as-prepared and heat-treated samples can be visualised in Figure 4. In the case of the *Gel* fibre web, a quite increase crystallinity is confirmed through the intense and narrow diffraction maxima. The fibrous template subjected to a three-cycle deposition is covered with a mixture of dicalcium phosphate dihydrate (CaHPO_4_·2H_2_O), which is commercially called brushite, with a monoclinic structure (ICDD 00-072-0713) and calcium phosphate hydroxide (Ca_10_(PO_4_)_6_(OH)_2_), known as hydroxyapatite, with hexagonal symmetry (ICDD 00-072-1243); the first phase is a majority, while the second one is a minority (Figure 4a). The addition of BT nanoparticles to the precursor solution changes to a certain extent the thermodynamics of the chemical reactions, since the ratio between the two mentioned CPs is reversed such that hydroxyapatite becomes quantitatively predominant; this can be attributed to a slight modification of pH, with significant repercussions on the entire process.

Going to the mineral composites achieved after the thermal treatments (Figure 4b), the temperatures of 800 and 1000 °C lead to tetragonal (ICDD 00-009-0346) calcium pyrophosphate (Ca_2_P_2_O_7_), while its growth to 1200 °C generates a combination of major orthorhombic (ICDD 00-009-0345) calcium pyrophosphate, and minor monoclinic (ICDD 00-029-0359) tricalcium phosphate (Ca_3_(PO_4_)_2_), which is in good agreement with our previous work [17]. The BT amount is again too small to be detected by the X-ray diffractometer.

It has already been reported that calcium pyrophosphate represents a promising candidate in the field of tissue engineering. Thus, the influence of doping on calcium pyrophosphate phase transformation, bulk densification, as well as cell proliferation was studied, showing a great potential for use in biomedical applications [57]. Calcium pyrophosphate powder was also integrated in chitosan or chitosan–gelatine matrices, which enhanced the mechanical properties and accelerated the mineralisation process, the derived systems being suitable for bone substitutes [58]. Calcium pyrophosphate was likewise composited with Ti-13Nb-13Zr alloy, demonstrating excellent mechanical and corrosion properties, high biocompatibility and bioactivity and, at the end, potential for orthopaedic implants [59]. Not least, the resorption simulation showed that the rate in the case of calcium pyrophosphate is lower than that for tricalcium phosphate and hydroxyapatite, which was associated with a weaker protonation of (P_2_O_7_)^4−^ ions during ceramic dissolution [60].

**Figure 5:**
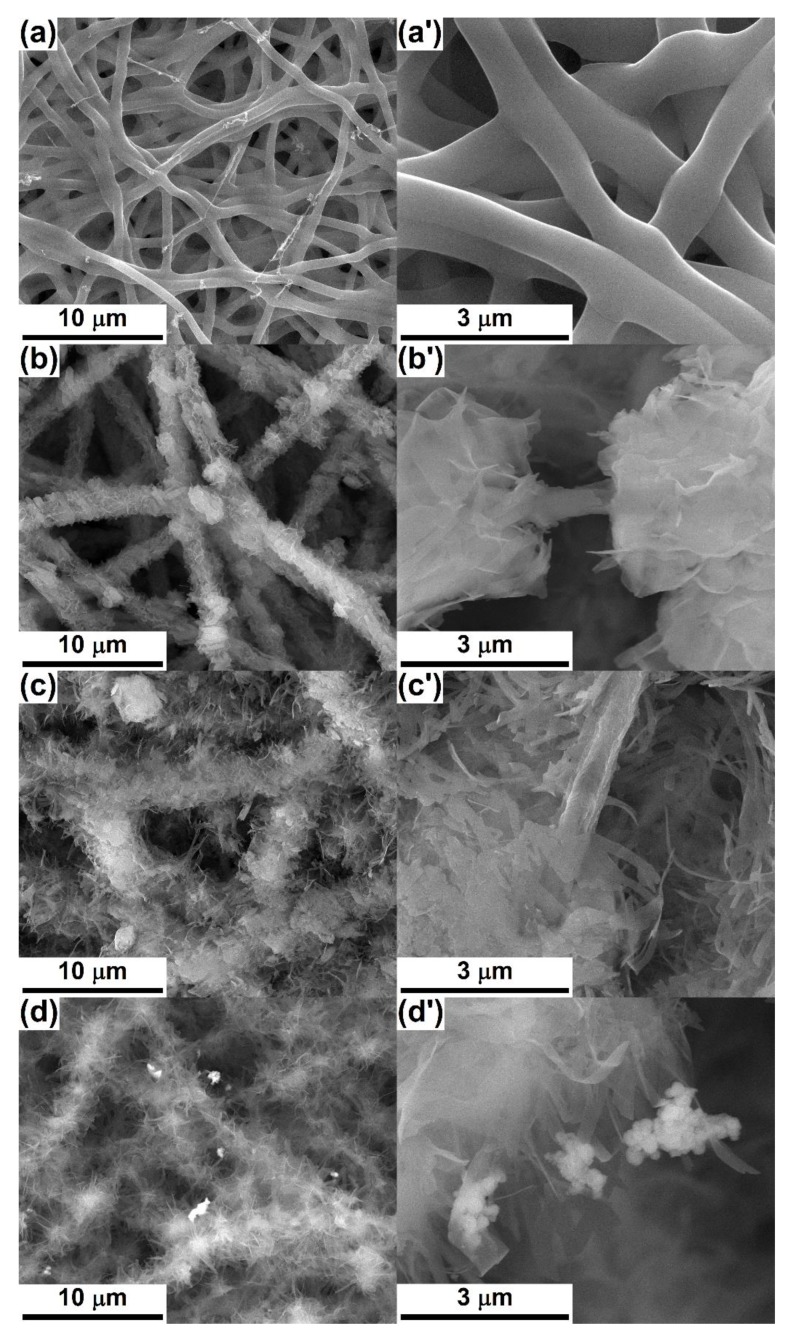
SEM images of: (**a**,**a**’) *Gel* fibre web, (**b**,**b**’) *Gel*-CPs-1c composite, (**c**,**c**’) *Gel*-CPs-3c composite and (**d**,**d**’) *Gel*-CPs-BT-3c.

**Figure 6:**
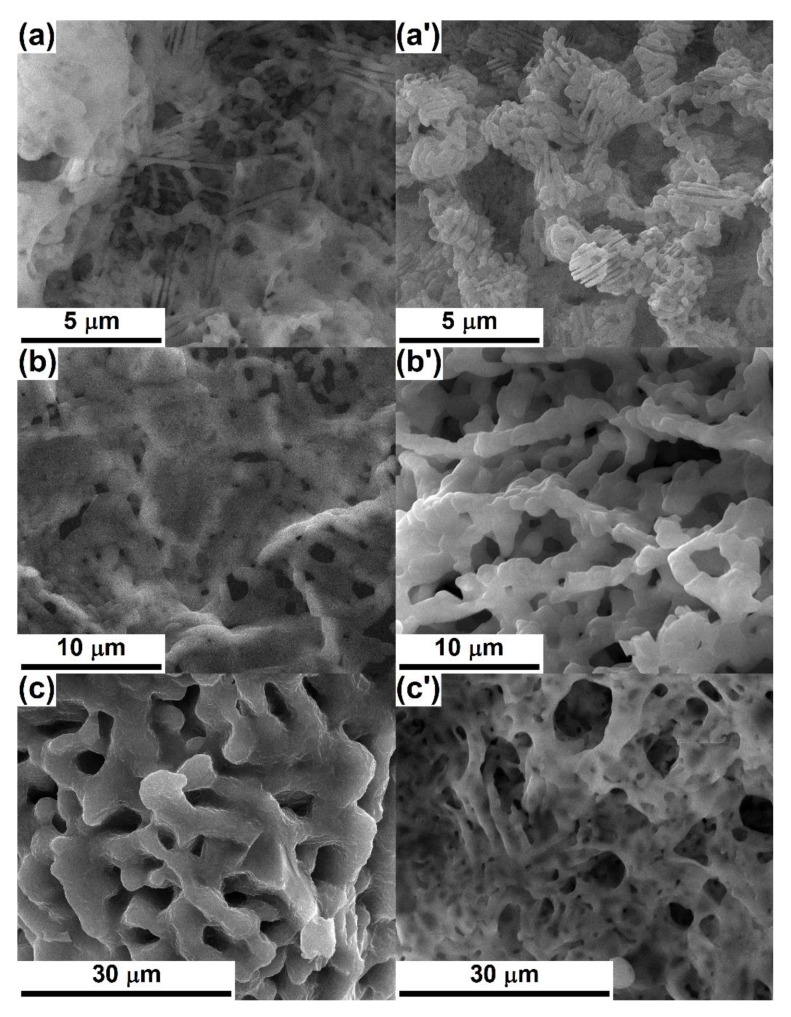
SEM images of *Gel*-CPs-3c composite thermally treated at: (**a**) 800 °C, (**b**) 1000 °C, and (**c**) 1200 °C and *Gel*-CPs-BT-3c composite thermally treated at (**a**’) 800 °C, (**b**’) 1000 °C, and (**c**’) 1200 °C.

The microstructure of the as-prepared and heat-treated scaffolds is evidenced in the SEM images centralised in Figure 5 and Figure 6. The bear *Gel* web is made of continuous nonwoven fibres, which are homogenous in thickness and randomly distributed in several tens of overlapping layers; their diameter falls within the range of 600–700 nm (Figure 5a,a’). After the loading process, the fibres get covered with CPs nanosheets packed in a loose way and radially arranged in relation to the main axis of the cylindrically shaped substrate. The final thickness of the whole structure is of 2–3 μm for *Gel*-CPs-1c (Figure 5b,b’) and 4–5 μm for *Gel*-CPs-3c (Figure 5c,c’), which means a mineral layer varying between 700 nm and 2.2 μm. When it comes to BT decoration, Figure 5d,d’, meaning SEM images based on the detected backscattered electrons, confirm its presence through the brighter domains, as well as physical attachment as aggregates of different sizes and shapes.

Figure 6 gives some examples of morphologies resulted for three deposition cycles and different temperature values. All the entities are three-dimensional and porous, showing the influence of the heat treatment on the grains dimension, shape, and interconnection, as well as pores ratio, size, geometry, and distribution within the volume. The samples thermally treated at 800 °C consist of elongated grains, even acicular sometimes, copying the spatial arrangement of the primary Gel fibres, but reduced in diameter (below 300 nm) due to polymer combustion and mineral phases shrinkage; such certain structures join in porous walls or bodies, which could contribute to the achievement of an appropriate mechanical resistance (Figure 6a,a’). As well, large pores, with sizes up to 4 μm and united in the form of a channel system that crosses the entire volume of the scaffold and seems suitable for the localisation of cells, are clearly visible. Going further to 1000 °C, it is obvious that the grains become larger (up to 2 μm), more rounded, and interconnected as a three-dimensional skeleton that is superposed over a network of branched pores; occasionally, between the main sustaining elements of this architecture, thin and soft filling panels occur, especially when BT is not involved, such a reinforcement being more prone to sustain device integrity in applications with frequent mechanical loads (Figure 6b,b’). With respect to the pores aspect, the trend can be divided in two directions, depending on the existence of the BT phase; thus, BC-CPs-3c (Figure 6b) exhibits smaller voids (below 2 μm) compared to the lower temperature counterpart (Figure 6a), which appeared due to the material shrinkage and could hinder the cells’ movement, while BC-CPs-BT-3c (Figure 6b’) preserves the empty areas and even expands them to 10 μm, as a consequence of material contraction only at the level of grains agglomeration, with the complete elimination of the elongated and parallel pores (Figure 6a’), which could provide a proper environment for the cells’ paths. Increasing the temperature to 1200 °C favours the material diffusion, leading to lower porosity, the appearance of bigger grains, and new bridges between them, which is equivalent to a densified material, with stronger links between components and beneficial effects on the mechanical properties (Figure 6c,c’). The scaffold without BT (Figure 6c) is the most massive, with interconnected pores having dimensions of maximum 7 μm and homogenously distributed within the volume, as a well-established circulation network. Contrariwise, the one with decoration (Figure 6c’) displays voids with a broad size distribution, different shapes, and variable degrees of binding; this look is the result of a preferential evolution and material displacement, which is in close connection with the local compositional and morphological particularities of the as-prepared sample. Considering the effect of *BT* nanoparticles decoration, this can be quantified starting from the differences in mineralogical composition both before and after the heat treatments (Figure 4), which are translated into a hindered densification and spongy character. This aspect can potentiate the biological response by at least two ways: on one hand, it offers an extended interface for the cells and, on the other hand, an external stimulation can be carried out by means of electrical/magnetic nature.

In this context, M. Ahmed et al. [61] approached the effect of the physical features of the fibrous scaffolds on cell migration and colonisation, concluding that the fibre diameter influences both cell morphology and alignment. Moreover, the importance of the fibres’ orientation for guided bone repair and reconstruction was also approached [62,63]. A comparison with our previously published articles on the same topic, but employing bacterial cellulose as a template, leads to the following statements: the first one addresses a material composed of BT grains interconnected in a branched structure and decorated with small clusters of CPs particles [16], the second describes highly porous three-dimensional architectures consisting of calcium pyrophosphate grains that sometimes bind in micro-sized walls [17], while the third one reports about buchwaldite and a hydroxyapatite-based scaffold made of a mixture of linked grains and rods or with trabecular aspect [18]. All these confirm the possibility of composition and morphology tuning via template selection, precursor combination, loading degree, and thermal parameters.

**Figure 7:**
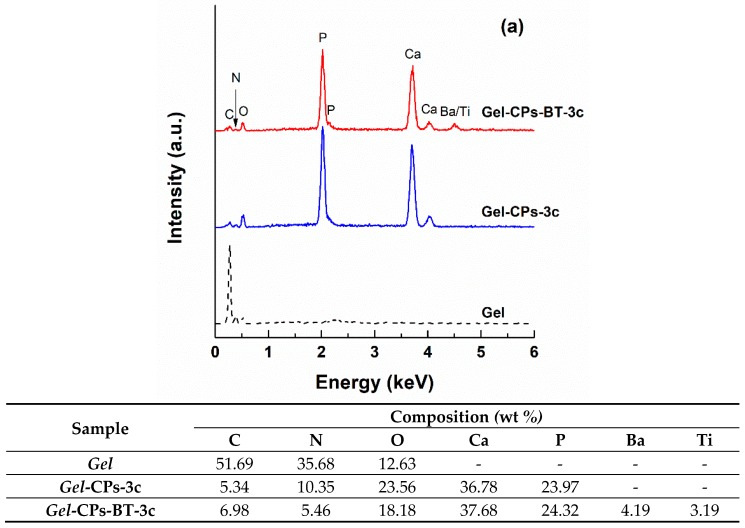

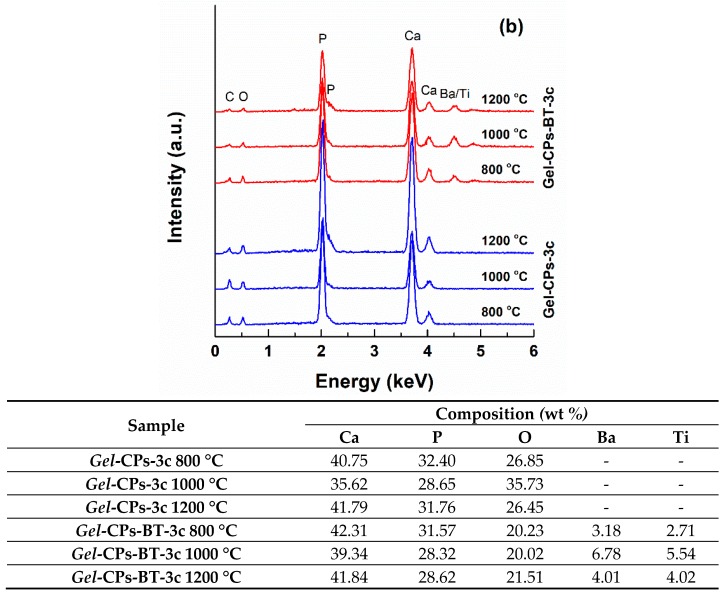
Energy-dispersive X-ray spectroscopy (EDX) spectra and corresponding elemental compositions of the obtained materials: (**a**) before the thermal treatments and (**b**) after the thermal treatments.

The evolution of the elemental composition during the loading/decoration procedures, as well as the situation of the thermally treated samples are highlighted in Figure 7. If *Gel* fibres consist of C, N, and O, these signals are shielded by the mineral coating that exceeds 700 nm, resulting in intense maxima for Ca and P, which is attributed to CPs, and weak peaks for Ba and Ti, which are assigned to BT (Figure 7a). Indeed, the detection of BT through this investigation method was possible only when recording the generated signals on an area previously validated as containing nanoparticle aggregates; otherwise, it is quite difficult to obtain a detectable contribution of this phase in the EDX spectrum, since the quantitative ratio between BT and CPs is extremely reduced even though 0.5 g of BT powder was added to (NH_4_)_2_HPO_4_ solution (only a small part of this amount was physically attached to CPs nanosheets). The other set of EDX spectra (Figure 7b) confirms the evanescence of the polymeric template (residual signals could come from the carbon tape employed for fixing the sample before analysis or adsorbed molecules of nitrogen from the surrounding atmosphere), together with the presence of the expected elements (Ca, P, Ba, Ti, and O) belonging to the scaffolds without or with BT nanoparticles decoration. Referring to the associated compositional data extracted from the previously discussed EDX spectra, it is obvious that all the values, as well as the differences from one specimen to another, falls within the detection limits of the EDX probe, confirming the reproducibility of the developed synthesis route.

**Figure 8:**
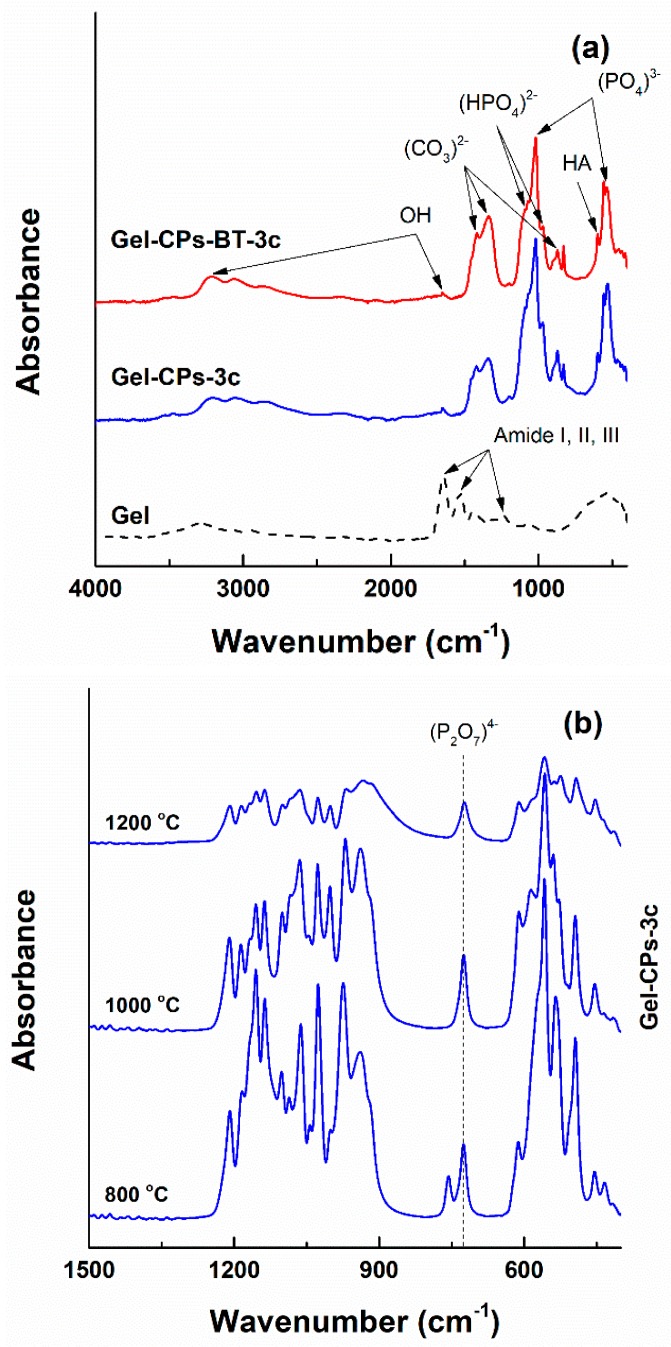

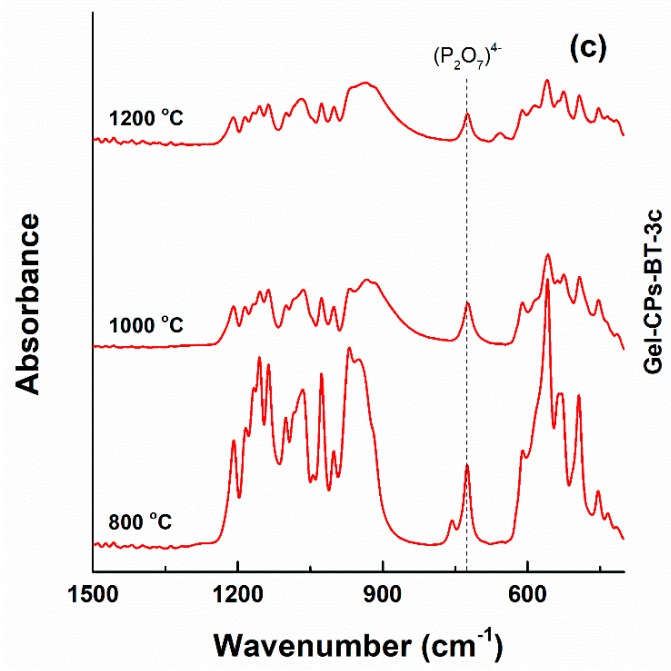
FTIR spectra of the obtained materials: (**a**) before the thermal treatments and (**b**,**c**) after the thermal treatments.

The way in which the formerly identified elements are linked in chemical groups can be analysed using the FTIR spectra (Figure 8). The specific absorption bands of Gel template are placed in the amide band region: amide I at 1646 cm^−1^, amide II at 1538 cm^−1^, and amide III at 1239 cm^−1^ [64]. Going to the coated fibres, the corresponding curves contain identification elements for an (HPO_4_)^3−^ group coming from brushite at 1090, 1071, and 970 cm^−1^ [65], a (PO_4_)^3−^ group belonging to hydroxyapatite at 1019, 558, and 537 cm^−1^ [66,67,68], a (CO_3_)^2−^ group as representative for the carbonated species or adsorbed carbon dioxide at 1417, 1338, and 871 cm^−1^ [69] and an (OH)^−^ group as an indication of the hydrated entities at 3213 and 1649 cm^−1^ [65] (Figure 8a). Detailing, the band centred at 871 cm^−1^ is a proof either of an A-type carbonated hydroxyapatite ((CO_3_)^2−^ group substituting (OH)^−^ group) or of a labile carbonate ((CO_3_)^2−^absorped on the surface of apatite crystals or separated carbonate phase present with apatite crystals) [69,70]. The sharp band looming at 829 cm^−1^ could be related to additional vibrations of the bond between a metallic cation and (OH)^−^ group [68]. Moreover, the emergence of a maximum at 599 cm^−1^ is a specific fingerprint of hydroxyapatite [71]. The vibrational band typical of the Ti–O bond, which is located somewhere in the range 450–550 cm^−1^, is hidden by the more intense contributions of the (PO_4_)^3−^ group [16,72,73]. The FTIR spectra registered on the samples subjected to heat treatments exhibit a well-defined individual band with a maximum around 720 cm^−1^ (Figure 8b); this is a mark of the vibrational characteristics of the (P_2_O_7_)^4−^ group, which was formed by a condensation reaction between two (HPO_4_)^2−^ groups at high temperatures [17,74].

## 4. Conclusions

The present work describes the route for obtaining mineral scaffolds based on calcium phosphates and barium titanate, with clinical applicability in the field of hard tissue engineering. In order to ensure an adequate microstructure regarding pores size, shape, and arrangement, suitable for cell adhesion, proliferation, and differentiation, a polymeric template in the form of fibrous webs was used. Gelatine fibres achieved by electrospinning were loaded with calcium phosphates and then decorated with barium titanate nanoparticles, which were both performed under ultrasound irradiation. The resulting composites were transformed into mineral scaffolds by heat treatment in different experimental conditions, so that we proposed several types of implantable materials with varied morphologies. The mineralogical composition undergoes a transition due to the heat treatments from a mixture of brushite and hydroxyapatite to calcium pyrophosphate, while the microstructure mimics the fibres’ layout for the lowest temperature and individualises as strengthened porous three-dimensional structures for the higher ones.

The future research will be dedicated to an extended biological evaluation of all developed materials, both from the perspective of the cell cultures response at different testing intervals, as well as the additional influence of a controlled external electric/magnetic field on the local amplification of the healing process.
